# Validity and feasibility of a satellite imagery-based method for rapid estimation of displaced populations

**DOI:** 10.1186/1476-072X-12-4

**Published:** 2013-01-23

**Authors:** Francesco Checchi, Barclay T Stewart, Jennifer J Palmer, Chris Grundy

**Affiliations:** 1 Faculty of Infectious and Tropical Diseases, London School of Hygiene and Tropical Medicine, Keppel St, London WC1E7HT, United Kingdom; 2 Faculty of Public Health and Policy, London School of Hygiene and Tropical Medicine, Keppel St, London WC1E7HT, United Kingdom

**Keywords:** Population, Estimation, Refugee, Internally displaced person, Humanitarian, War, Displaced, Satellite imagery, Remote sensing

## Abstract

**Background:**

Estimating the size of forcibly displaced populations is key to documenting their plight and allocating sufficient resources to their assistance, but is often not done, particularly during the acute phase of displacement, due to methodological challenges and inaccessibility. In this study, we explored the potential use of very high resolution satellite imagery to remotely estimate forcibly displaced populations.

**Methods:**

Our method consisted of multiplying (i) manual counts of assumed residential structures on a satellite image and (ii) estimates of the mean number of people per structure (structure occupancy) obtained from publicly available reports. We computed population estimates for 11 sites in Bangladesh, Chad, Democratic Republic of Congo, Ethiopia, Haiti, Kenya and Mozambique (six refugee camps, three internally displaced persons’ camps and two urban neighbourhoods with a mixture of residents and displaced) ranging in population from 1,969 to 90,547, and compared these to “gold standard” reference population figures from census or other robust methods.

**Results:**

Structure counts by independent analysts were reasonably consistent. Between one and 11 occupancy reports were available per site and most of these reported people per household rather than per structure. The imagery-based method had a precision relative to reference population figures of <10% in four sites and 10–30% in three sites, but severely over-estimated the population in an Ethiopian camp with implausible occupancy data and two post-earthquake Haiti sites featuring dense and complex residential layout. For each site, estimates were produced in 2–5 working person-days.

**Conclusions:**

In settings with clearly distinguishable individual structures, the remote, imagery-based method had reasonable accuracy for the purposes of rapid estimation, was simple and quick to implement, and would likely perform better in more current application. However, it may have insurmountable limitations in settings featuring connected buildings or shelters, a complex pattern of roofs and multi-level buildings. Based on these results, we discuss possible ways forward for the method’s development.

## Introduction

Currently, an estimated 43 million people worldwide are forcibly displaced due to armed conflict or other crises; of these, about 10 million are refugees and the remainder internally displaced persons (IDPs) [1]. Knowing the size of the displaced population in a given site is critical to interpret indicators (e.g. crude death rate, severe acute malnutrition prevalence, sanitation coverage), effectively allocate resources (e.g. curative health services, vaccines and other preventive interventions, food, non-food items, etc.) and plan mitigation measures to address added pressure on natural resources due to the arrival of the displaced population [2]. Quantifying the number of displaced people is a mandated function of the United Nations High Commissioner for Refugees (UNHCR) [3] and a common Sphere Standard for Initial Assessment [4]. However, in the acute emergency phase of displacement reliable population figures are often not available, and asking the refugee or IDP leadership to perform its own census may result in bias [5]. Population estimation is not sufficiently prioritised, and expertise in ground-based estimation rarely available in the first days or weeks of displacement events, particularly given that existing statistically robust methods require fairly complex sampling and analysis [6-10]. Moreover, many current guidelines for relief agencies recommend methods that are not validated or are based on convenience samples [11]. Decreasing humanitarian access [12] means that many displaced populations are intermittently accessible: failure to document the size of these populations may lead to their neglect by governments, relief agencies and humanitarian funding mechanisms.

Satellite imagery is increasingly available and has seen expanding application in recent emergencies for regional level mapping, site planning and vulnerability or damage assessments. Satellite imagery has also seen some use in non-emergency settings to estimate population sizes [13-17]. Very high spatial resolution (VHSR) images, defined by resolution <4 m, are of particular interest for the data needs of the humanitarian sector, since they enable visualisation of individual residential structures such as tents, huts or other buildings. VHSR sensors are currently onboard several orbiting satellites, take frequent images and can be tasked on request to commission imagery of particular sites and time points of interest. Several exploratory projects using VHSR imagery for mapping IDP or refugee settlements have taken place. Multiple groups have analysed camps in Tanzania and Darfur and showed that algorithms for automated counting of residential structures can achieve reasonable precision compared to manual methods [18-21]. The Operational Satellite Applications Programme of the United Nations Institute for Training and Research and the European Union Joint Research Centre conducted remote assessments of shelters and bomb damage during the final phase of the war in Sri Lanka in 2009 (see http://www.unitar.org/unosat/ and Kemper et al. [22]). Since 2008, Metria, a Swedish company, has supported UNHCR in performing repeat manual counts of shelters and buildings in the Afgooye IDP corridor in Somalia, which are combined with UNHCR ground data on population (see http://www.metria.se/Startpage/News1/News-1/). Despite the above advances, we could find only one study [23] that sought to validate an imagery-based population estimate against robust ground population estimates; furthermore, while this study achieved high accuracy (1% difference between the estimates), the remote estimate used ground data on population density.

In this study, we sought to develop a relatively simple, remote analysis method for estimating IDP and refugee populations based on VHRS imagery. We aimed to validate the method in a variety of sites featuring different settlement patterns, by comparing estimates to available reference population figures derived by gold standard methods on the ground (see below).

## Methods

### Overview of the method

The method we tested consists of the following three steps (see below): (i) manually count all residential structures visible on the satellite image and falling within the site’s boundaries; (ii) review the published and unpublished literature, including web sources, for estimates of the number of people per structure (structure occupancy) in the site or in similar sites within the same crisis region; and (iii) multiply the structure count by the average estimate of structure occupancy from the literature to obtain a population estimate.

### Selection of study sites

We tested the method retrospectively in 11 sites (Table 1), chosen because they featured (i) an IDP or refugee population; (ii) a reference “gold standard” estimate of population size obtained through census, exhaustive registration or demographic surveillance (i.e. methods that are well-documented and considered robust), and supported by adequate documentation (namely a report or protocol providing information on how the gold standard estimate was arrived at); and (iii) at least one VHSR satellite image covering the entire site, with cloud cover <10%, resolution <70 cm and taken within one month (stable sites) or one week (acute emergency situations) before/after the date of the reference population estimate. We aimed to include a range of settlement patterns from different areas of the world. We excluded a priori urban settings with multi-storey buildings as it was recognised from the start that these would not be suitable for the simple manual counting approach being tested, due to the difficulty in assessing number of storeys from a satellite image. In practice the selection of sites was heavily constrained by very limited options considering our simultaneous requirements for available imagery and a reference estimate.

**Table 1:** Description of study sites

**Site name**	**Country (region/city)**	**Settlement type**	**Crisis**	**Reference population estimate**	**Source of reference estimate**	**Date of reference estimate**	**Date of satellite image**	**Notes**
Kutupalong	Bangladesh (Cox’s Bazar District)	Refugee camp	Rohingya refugees from Rakhine State, Myanmar	11,047	UNHCR registration (unpublished data)	31 Dec 2009	29 Jan 2010	Also analysed a makeshift camp (estimated population 20–30,000) surrounding formal refugee camp.
Breidjing	Chad (Ouaddaï Region)	Refugee camp	Sudanese refugees from Darfur	26,770	UNHCR registration (unpublished data)	31 Dec 2005	30 Jan 2006	Médecins Sans Frontières (MSF) demographic surveillance estimated 27,500 people [24].
Farchana	Chad (Ouaddaï Region)	Refugee camp	Sudanese refugees from Darfur	19,070	UNHCR registration (unpublished data)	31 Dec 2004	11 Oct 2004	MSF demographic surveillance estimated 16 250 people [24]. >1 month between image and analysis dates.
Bambu	Democratic Republic of Congo (North Kivu Province)	IDP camp	Insecurity and attacks against civilians	5871	UNOPS Data Center for IDP demographic surveillance [25]	31 Jan 2010	29 Jan 2010	Analysis date chosen to coincide with population verification exercise.
Mugunga III	Democratic Republic of Congo (North Kivu Province)	IDP camp	Insecurity and attacks against civilians	1969	UNOPS Data Center for IDP demographic surveillance [25]	31 Jan 2010	29 Jan 2010	Analysis date chosen to coincide with population verification exercise.
Sherkole	Ethiopia (Benishangul-Gumuz Region)	Refugee camp	Southern Sudanese refugees, mainly from Blue Nile state	13,958	UNHCR registration (unpublished data)	31 Dec 2006	8 Nov 2006	>1 month between image and analysis dates.
Shimelba	Ethiopia (Tigray Region)	Refugee camp	Eritrean refugees	13,043	UNHCR registration (unpublished data)	31 Dec 2006	29 Oct 2006	>1 month between image and analysis dates.
Champs-de-Mars	Haiti (Port-au-Prince metropolitan area)	Informal IDP camp	Earthquake	23,214	Médecins Sans Frontières/Epicentre census [26]	22 Apr 2010	30 Apr 2010	
Delmas 24, Sollino, Fort National	Haiti (Port-au-Prince metropolitan area)	Urban neighbourhoods with mixture of residents and informal IDPs	Earthquake	39,349	Médecins Sans Frontières/Epicentre census [26]	13 May 2010	11 May 2010	
Kakuma	Kenya (Turkana District)	Refugee camp	Southern Sudanese, Somali and other refugees	90,457	UNHCR registration (unpublished data)	31 Dec 2006	14 Jan 2007	
Bairro Esturro	Mozambique (Beira municipality)	Urban neighbourhood with few IDPs	Residual displacement from civil war	9523	T-square method estimate by Epicentre [8]	15 Aug 2004	15 Aug 2004	Analysed only section of Bairro Esturro included in reference study. A census took place in Sep 2003 and yielded a similar estimate (9479).

Eight sites were in Sub-Saharan Africa. Six were UNHCR-supported refugee camps (selected based on information from UNHCR that the camps in question had exceptionally reliable registration procedures in place), three were IDP camps and two were urban neighbourhoods with a mixture of residents and IDPs (Table 1). Sites ranged in population from 1,969 to 90,457 (median 13,958). Further descriptions are provided in the Additional file 1.

### Imagery acquisition and manual structure count

We obtained free of charge imagery of Kutupalong camp from the US Department of State, and sourced the remainder from archives of commercial resellers. The majority of images were generated by the WorldView-1, WorldView-2 and Quickbird satellites, which provide a multi-spectral image resolution of 65 cm. The Haiti images came from the GeoEye-1 satellite, with a resolution of 50 cm. A mixture of delivery and spectral options was selected, including 3 band pan-sharpened, 4 band pan-sharpened and 4 band bundle (4 multi-spectral bands and 1 panchromatic band). Only one candidate image fulfilling our criteria was available for each site, and therefore the chosen resolution and image options were dictated by commercial offer.

We first explored the image, pan-sharpening images that were supplied as a 4 band bundle, experimenting with basic histogram stretching, and displaying images in true and false colour where possible. The exact camp outline or section of the image to be counted was defined along with the typology of structures and residential layouts present in the site. We then overlaid gridlines of 200 m onto the image, which allowed us to organise the count. After trialling the count procedure on 2–3 squares, analysts manually marked structures in each grid square, proceeding systematically from the top-left corner of the image. We viewed each square in both true and false colour (where available) so as to maximise the contrast between structures and other land features. We classified observed structures into categories that were appropriate for the site (e.g. traditional huts, tents, large buildings, etc.). Counting was done in duplicate, with analysts blinded to each other’s results and to the reference population estimate. Ahead of the count, the two analysts discussed the image and attempted to agree on which types of structure to mark as residential and on the definition of large buildings (typically these were unusually shaped and sized polygons occurring at the periphery or within defined areas of the site, strongly indicative of relief warehouses, schools, places of worship and government facilities). Each analyst then decided by eye which category to classify each structure into. Counting was carried out in ArcGIS 10.1 (Esri, Redlands, CA, USA).

### Structure occupancy estimate review

We sought reports published during the 10y prior to each site’s analysis date, and containing an estimate of *mean* residential structure occupancy or mean household size within the site itself; similar sites within the same crisis region (e.g. other Sudanese refugee camps in eastern Chad; IDP camps in the eastern DRC); or for the population currently living in the site but before displacement. We included such reports in the analysis if the full text was available and contained primary data on occupancy; and, for non-camp, urban sites, if the report reflected urban populations only (e.g. for post-earthquake sites in Port-au-Prince, we excluded estimates of occupancy from rural sites).

For each site, we carried out a systematic search for the above reports, targeting both published and grey literature. The search strategy was designed to be feasible for an analyst with moderate skills working under emergency timelines to generate a population estimate. Details of the search strategy are provided in the Additional file 1.

So as to deal with multiple estimates for the same site and quantify the amount and quality of information available, we attributed an “information score” to each report, computed based on a hierarchy of evidence built along three attributes (how representative the report was of the site’s population at the time point analysed; how robust the data collection method was; and whether the estimate reflected the size of households as opposed to residential structures), as shown in Table 2. We computed the information score for each report by multiplying points for each of the three attributes. Each score is therefore out of a possible 1000 maximum. For each site, we also calculated an “amount of information index” by summing scores of all available reports.

**Table 2:** Hierarchy of evidence used to assign information scores to structure occupancy reports

**Category**	**Points**
**Attribute 1: How representative of the site’s population and analysis time-point is the report likely to be?†**	
Report from the site itself during the current crisis, as defined by data collection having taken place within 3y of the date of analysis	10
Report from site(s) within the same crisis region and during the current crisis (data collection within 3y of data of analysis)	8
Report from the site itself or similar sites within the same crisis region, but from previous crisis periods, as defined by data collection having taken place prior to 3y but within 10y of the date of analysis	4
Report from the population currently living in the site but reflecting pre-displacement conditions (e.g. while residing in the country of origin), with data collected within 10y of the date of analysis	2
**Attribute 2: How robust is the method for data collection on which the estimate is based?**	
Census, systematic registration exercise or ongoing demographic surveillance	10
Large (>200 households for simple/systematic random sampling, >400 households and >20 clusters for cluster sampling) sample survey or cross-sectional population sample with no obvious technical flaw(s)	8
Other sample survey or cross-sectional population sample	6
Estimate from rapid assessment, site visit or review of programmatic data	4
Anecdotal or unsubstantiated estimate	1
**Attribute 3: Does the report quantify the mean occupancy of households or residential structures?**	
Residential structures	10
Households, but the household definition is consistent with one household = one structure (e.g. “people sleeping together under one roof”) or one household = one compound (e.g. ‘people sharing meals’) in settlements consisting of compounds or groups of structures demarcated by rings or fences visible on the satellite image	6
Households, and the household definition is either unclear or may not be congruous with that of a single structure	1

† If an occupancy estimate was computed based on a sample consisting of both the site and surrounding sites, with no breakdown of results by site, we calculated an average score for this attribute based on the proportion of the sample falling within the site. If this was not provided, we attributed 2 points (the minimum).

### Population estimation

To compute population estimates, we used a simple approach that may be appropriate for the likely skills set of a remote imagery team and that may facilitate interpretation by agencies. This consisted of multiplying the following quantities: (i) the mean of the two independent structure counts; and (ii) the weighted mean of the available occupancy estimates for the site, with weights given by the information score associated with each occupancy estimate (i.e., where n is the total number of occupancy estimates available, x_i_ is the estimate from report i and w_i_ is the score of report i).

## Results

### Structure counts

Differences in the two independent counts were moderate for residential structures (Table 3), although strong discrepancies were noted for Delmas 24 and Bairro Esturro. There was far more discrepancy in counts for non-residential structures: no agreement was reached ahead of analysis on which non-residential structures to count, as these did not have any influence on the population estimate; typically, one of the two analysts made a systematic decision to count or omit very small structures, e.g. probable latrines or showers. There was no apparent correlation between percent agreement and the number of structures in the site (data not shown).

**Table 3:** Results of duplicate structure counts, by site and assumed type of structure

**Site name**	**Residential structures (used for population estimate)**			**Other structures**			**Total structures**		
	**Count 1**	**Count 2**	**Difference†**	**Count 1**	**Count 2**	**Difference†**	**Count 1**	**Count 2**	**Difference†**
Kutupalong	371‡	371‡	0 (0%)	440	444	4 (0.9%)	811	815	4 (0.5%)
Breidjing	5423	6208	785 (12.6%)	564	903	339 (37.5%)	5987	7111	1124 (18.8%)
Farchana	4181	3466	715 (17.1%)	493	900	407 (45.2%)	4674	4366	308 (6.6%)
Bambu	1501	1380	121 (8.1%)	41	50	9 (18.0%)	1542	1430	112 (7.3%)
Mugunga III	588	518	70 (11.9%)	2	129	127 (98.4%)	590	647	57 (8.8%)
Sherkole	2643	2746	103 (3.8%)	251	217	34 (13.5%)	2894	2963	69 (2.3%)
Shimelba	2500	2604	104 (4.0%)	741	408	333 (44.9%)	3241	3012	229 (7.1%)
Champs-de-Mars	2169	2552	383 (15.0%)	70	0	70 (100.0%)	2239	2552	313 (12.3%)
Delmas 24, Sollino, Fort National	2929	4849	1920 (39.6%)	430	472	42 (8.9%)	3359	5321	1962 (36.9%)
Kakuma	16,690¶	11 342	1137 (9.1%)	2661¶	2305	904 (39.2%)	19,351¶	13 647	233 (1.7%)
Bairro Esturro	1643	1194	449 (27.3%)	242	222	20 (8.3%)	1885	1416	469 (24.9%)

Both absolute and relative percent differences (in parentheses) between the two counts are presented.

† All relative differences are the absolute value of the difference in counts divided by the larger of the two counts.

‡ In Kutupalong formal camp all visible residential structures were in fact large multi-household sheds, resembling barracks.

¶ Count 2 covered only a sub-section of Kakuma camp. Count 1 for this same section was 12 479 residential, 1401 other and 13 880 total structures.

While seven camps were chacterised by a predominant residential structure type, in four sites, Farchana, Shimelba, Delmas 24 and Kakuma, residential structures appeared to be a mix of different types, usually a combination of traditional huts, tents and small buildings. In these settings there were considerable discrepancies between analysts as regards which category each structure was placed in. In Farchana camp, the first and second counts identified 3049 small huts and 1132 tents versus 2386 small huts and 1080 tents. In Shimelba camp, counts identified 373 traditional huts and 2127 houses versus 432 traditional huts and 2172 houses; while in Delmas 24 1354 tents and 1874 houses were identified by the first count versus 1575 tents and 2975 houses by the second (in Kakuma only one category was used during counting).

In Kutupalong makeshift camp (located all around the official camp), 3708 residential structures were additionally counted (not shown in Table 3 as a single analyst did the count for this site).

### Issues encountered during image analysis

**Figure 1:**
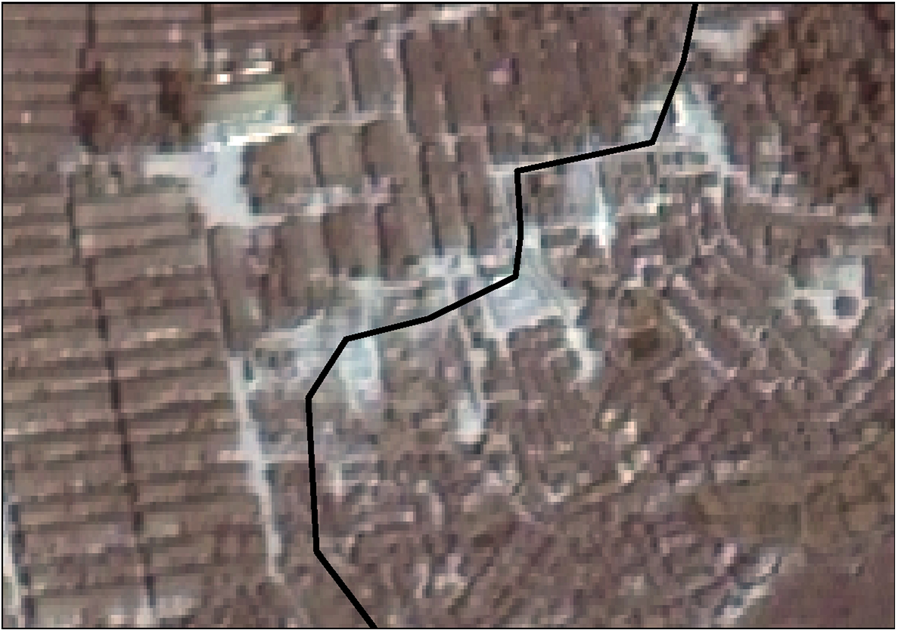
**Sections of Kutupalong official (left of black line) and makeshift (right of black line) camps. **Image copyright DigtalGlobe.

As shown in Figure 1, Kutupalong official and makeshift camps were adjacent. The former featured only one residential structure type (long, easily distinguishable multi-household sheds). The latter was comprised of smaller slum-like dwellings, clumped very close together with minimal street separation and a chaotic layout. The image did not clearly allow individual huts to be identified, and surrounding vegetation often looked similar to these structures.

**Figure 2:**
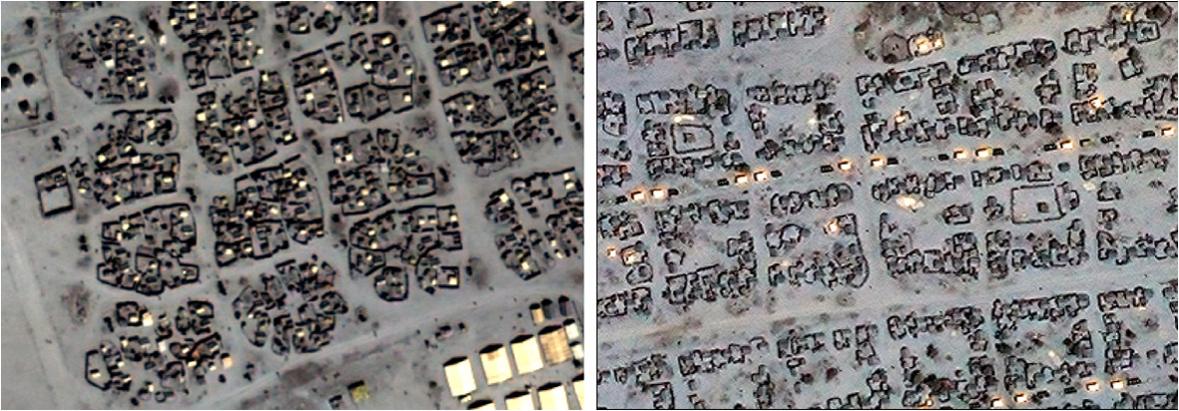
**Sections of Breidjing (left) and Farchana (right) camps. **Image copyright DigtalGlobe.

Breidjing camp contained a fairly consistent block layout (Figure 2), but within these blocks structures were difficult to identify, as walls or fences around dwellings created a light-shade contrast that blended with that of surrounding features. Farchana camp was also mostly organised in blocks (Figure 2), but with a more predictable structure of four dwellings per block side and a less extensive network of fences, yielding a cleaner image to view.

**Figure 3:**
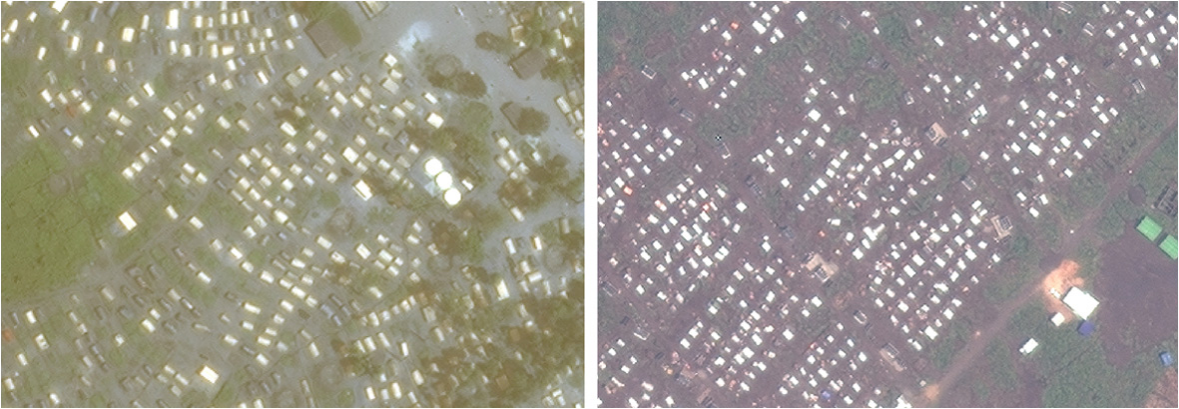
**Sections of Bambu (left) and Mugunga III (right) camps. **Image copyright DigtalGlobe.

Bambu camp featured mostly tent-like structures of variable size (Figure 3); these were easily distinguishable from other land features, but tree cover was considerable (though most tree-shaded dwellings seemed at least partly visible). Mugunga III had a similar layout but a visually messier image (Figure 3), with small white-colour areas difficult to visually classify as tents in current use, tents that may have been abandoned, debris from previous tents, or merely “noise” (pixels with no data).

**Figure 4:**
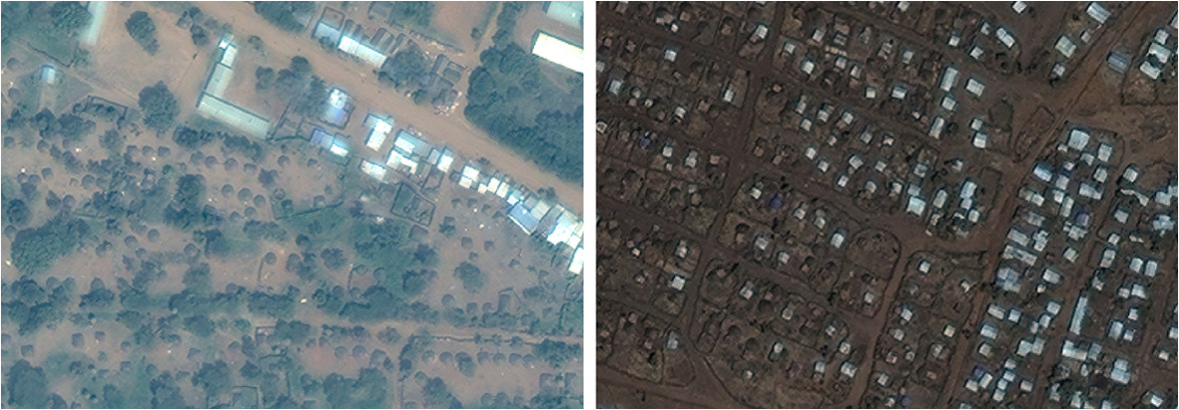
**Sections of Sherkole (left) and Shimelba (right) camps. **Image copyright DigtalGlobe.

Apart from an apparently commercial strip, Sherkole camp mainly consisted of traditional huts (Figure 4). The challenges were tree cover and in one section distinguishing huts from straw bales in fields. Counting of Shimelba camp (Figure 4) was also straightforward apart from two dense sections difficult to categorise as residential huts or market shops.

**Figure 5:**
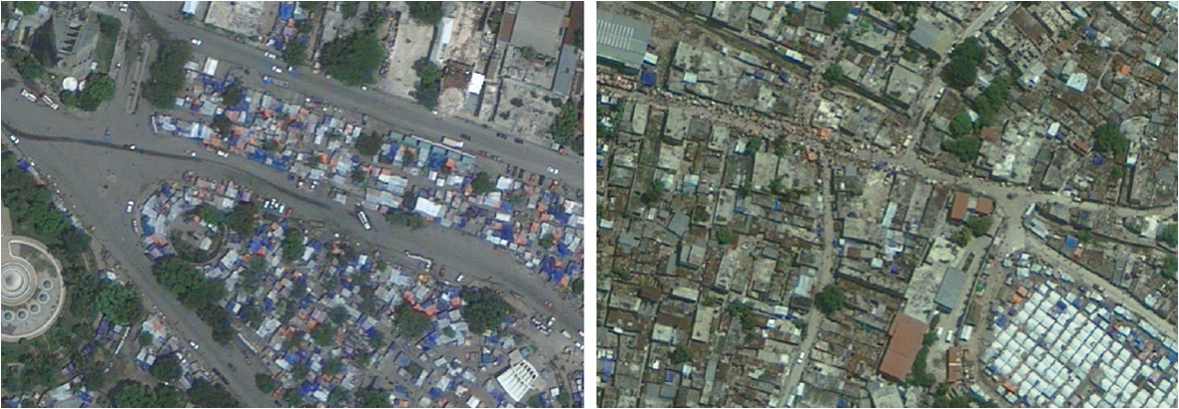
**Sections of Champs de Mars camp (left) and Delmas 24, Sollino and Fort National neighbourhoods (right). **Image copyright GeoEye Inc.

Champ de Mars featured very high-density tents and temporary roofing, with no demarcation between structures other than different roof colours (Figure 5). The site also contained considerable tree cover. Fort National, Sollino & Delmas 24 was a mix of tent camps (some organised and easily countable, others resembling Champ de Mars), buildings of various size and tents or tarpaulin sheets located among these buildings, presumably next to collapsed houses (Figure 5). We found it almost impossible to distinguish collapsed from standing structures and decide what constituted a single-household dwelling given the very high density network of roofs, chaotic layout and wide variety in building size, shape and colour.

**Figure 6:**
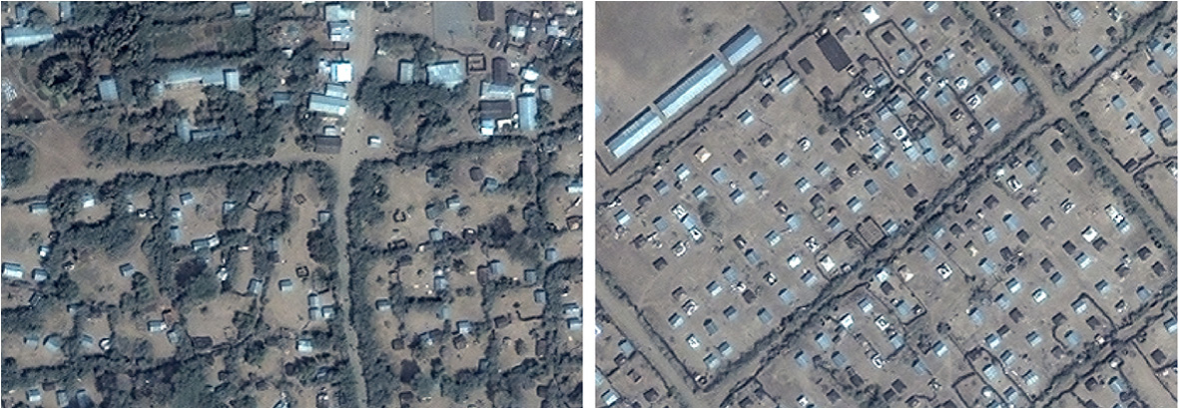
**Sections of Kakuma camp.** Image copyright DigtalGlobe.

Exceptionally large Kakuma camp was a mixture of tented areas, small rectangular dwellings, traditional or slum-like huts and closely packed buildings, including two or three commercial sections. Tent and residential hut sections of the camp (Figure 6, left) were straightforward to count, with town areas (right) being more challenging.

**Figure 7:**
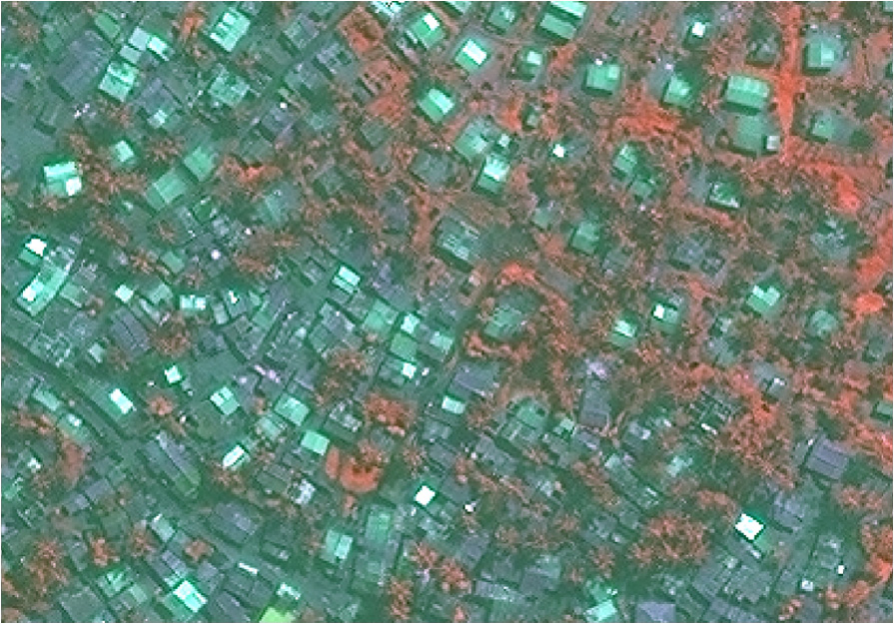
**Section of Bairro Esturro. **Image copyright DigtalGlobe.

The image for Bairro Esturro predominantly showed stand-alone dwellings that were only partly obscured by vegetation (Figure 7). However, the centre of the site (most of Figure 7) featured very high density habitation with no clear boundary between one dwelling and the next. Distinguishing dwellings was difficult initially, partly due to their colour similarity to the background.

### Structure occupancy estimates

The search strategy yielded few eligible reports, ranging from only one to 11 per site (Table 4). A total of 38 reports were eligible considering all sites (19 of these were used to populate occupancy estimates for multiple sites within the crisis region). A full listing is provided in the Additional file 1. Six of the 38 reports contained data on one of the sites collected within the past 3y; a further five referred to nearby similar sites within the past 3y; 16 referred to one of the sites or similar sites but at a time point 10-3y prior; while 11 were pre-displacement estimates. The vast majority (31/38) were household sample surveys, of which 16 were large according to our scoring criteria (Table 2). Most reports (28/38) provided an estimate of household size, either not defined or with a definition that may not have been consistent with occupying the same structure (e.g. “people eating together”). Only 5/38 reports estimated the mean structure occupancy, while a further five estimated household size but defined in a way that was consistent with a residential structure. None of the reports provided information on occupancy by type of structure.

**Table 4:** Population estimates based on remote imagery analysis and the reference method

**Site name**	**Results from imagery-based method**			**Reference population estimate**	**Absolute difference (relative%)†**	**“Amount of information“ index (number of reports)**
	**Mean structure count**	**Weighted mean structure occupancy**	**Estimated population**			
Kutupalong	371	6.5 x 5‡ = 32.5	**12,058**	**11,047**	+1011 (+9.2%)	789 (6)
Breidjing	5816	6.0	**34,896**	**26,770**	+8126 (+30.4%)	800 (3)
Farchana	3824	6.0	**22,944**	**19,070**	+3874 (+20.3%)	808 (3)
Bambu	1441	5.3	**7637**	**5871**	+1766 (+30.1%)	452 (11)
Mugunga III	553	5.4	**2986**	**1969**	+1017 (+51.7%)	436 (11)
Sherkole	2695	3.1	**8355**	**13,958**	−5603 (−40.1%)	16 (1)
Shimelba	2552	4.7	**11,994**	**13,043**	−1049 (−8.0%)	60 (1)
Champs-de-Mars	2361	5.3	**12,513**	**23,214**	−10,701 (−46.1%)	880 (5)
Delmas 24, Sollino, Fort National	3889	5.3	**20,612**	**39,349**	−18,737 (−47.6%)	880 (5)
Kakuma	16,690¶	5.3	**88,457**	**90,457**	−2000 (−2.2%)	428 (4)
Bairro Esturro	1419	6.3	**8940**	**9523**	−583 (−6.1%)	252 (7)

† Relative difference computed as the difference between estimates divided by reference estimate.

‡ Average of 5 families per shed as reported by Feeny [27].

¶ Only count 1 was considered.

### Population estimates and validation

The imagery-based method achieved a good degree of precision (relative difference compared to the reference population <10%) for Kutupalong, Shimelba, Kakuma and Bairro Esturro (Table 4). In Bairro Esturro, The T-Square method survey (a population estimation method that combines area sampling with average distance between structures and occupancy questionnaires [8]) on which the reference estimate was based yielded a figure of 1,685 residential structures with a mean occupancy of 5.3, while a government census done a year earlier had counted 1,828 structures with an occupancy of 5.1. We thus under-counted residential structures but balanced this with a higher occupancy estimate (Table 4). The estimate for Kutupalong makeshift camp (not included in Table 4 due to the absence of a gold standard reference estimate) was 24,102, compared to various estimates of 20,000 to 30,000 over the year 2010 [28].

A moderate precision of 10–30% was achieved for Breidjing, Farchana and Bambu. There was considerable over-estimation for Mugunga III (although modest in absolute terms), and severe under-estimation for Sherkole (for which only one, fairly implausible estimate of occupancy was available), and for the two Haiti sites. In Champs-de-Mars, 4542 shelters were counted by Médecins Sans Frontières census teams, with a mean occupancy of 5.1, while in Delmas 24 these figures were 8565 and 4.6 respectively, illustrating the extent of our under-count (Table 4).

### Efficiency

As shown in Table 5, the total person-time required to implement the method fully was reasonably consistent across sites, ranging from 16 to 42 person-hours, i.e. about 2 to 5 working days of a single analyst. Most person-time (47%) was devoted to searching for occupancy reports. Preparing images and counting structures took up 17% and 24% of the total person-time, respectively. Counting time per capita population was also fairly consistent, ranging from 0.19 to 0.51 hours per 1000 population (mean 0.30): accordingly, a site of 100,000 people might be projected to require about 30 hours.

**Table 5:** Person-time inputs (hours) for the various steps of the population estimation method, by activity and site

**Site name**	**Time per activity (person-hours)**						**Total person-hours**	**Counting time per 1000 population‡**
	**Obtain imagery**	**Prepare imagery†**	**Count structures**	**Occupancy data search**	**Review occupancy**	**Compute population**		
Kutupalong	1.75	7.50	4.50	4.75	0.50	0.25	19.25	0.41
Breidjing	2.75	1.75	5.00	7.50	0.25	0.25	17.50	0.19
Farchana	1.50	1.50	4.75	7.50	0.25	0.25	15.75	0.25
Bambu	1.25	3.00	1.25	15.25	0.75	0.25	21.75	0.21
Mugunga III	1.25	0.75	1.00	15.50	0.75	0.25	19.50	0.51
Sherkole	1.00	1.25	3.75	10.25	0.25	0.25	16.75	0.27
Shimelba	1.00	1.00	4.00	10.50	0.25	0.25	17.00	0.31
Champs-de-Mars	4.25	7.50	7.50	13.50	0.50	0.25	33.50	0.32
Delmas 24, Sollino, Fort National	4.50	6.75	12.00	13.00	0.50	0.25	37.00	0.30
Kakuma	2.75	8.50	20.75	12.25	0.25	0.25	44.75	0.23
Bairro Esturro	6.25	9.50	2.75	23.00	0.75	0.25	42.50	0.29

† Work required to prepare the image for counting, including pan-sharpening if needed, creating the camp outline and the 200 m grid.

‡ Based on reference population estimate. Only person-time for structure counting is considered, as all other activities are less dependent on the site’s population size.

## Discussion

To our knowledge, this is the first study to have evaluated the validity of IDP or refugee population estimation based on satellite imagery in a variety of different sites and phases of displacement. Our findings suggest that a remote analysis approach relying on manual counting of structures and published occupancy estimates can achieve reasonable precision in sites where individual structures are distinguishable and neither clouds nor vegetation pose a significant barrier to visual analysis.

The method’s performance on the whole suggests that, rather than referring to it as a valid approach, one could consider it “good enough” for certain purposes, assuming that no robust ground estimation is possible within the same timeframe. Specifically, while inaccuracy of up to 30% is probably unacceptable in post-acute emergency scenarios where resources for on the ground population tracking are present, we believe that for the purposes of initial planning (e.g. vaccination, distribution of food and non-food items, emergency water and sanitation provision), this level of inaccuracy is a substantial improvement over no information or guesswork, which might be the case if the site is inaccessible or if expertise in ground estimation cannot be sourced. However, the expected level of inaccuracy of the method would have to be explicitly emphasised when presenting this as an option for rapid estimation.

Remote analysis appears feasible in terms of human resources and financial inputs: in our study, it required 2–5 days, two analysts and, apart from salary and office expenditures, only minimal imagery procurement costs (15 to 25 USD per km^2^, though these costs would be somewhat higher if images were commissioned).

Visual analysis of the imagery was not overly complicated, despite most analysts in this study having no prior GIS skills. However, the visual quality and complexity of the image were critical determinants of both speed and accuracy of counting. While experienced spatial analysts may be able to improve image quality by using various techniques that enhance the visibility of features, we wished to evaluate use of the method by analysts with limited GIS skills, and thus refrained from making such improvements to the images. Moreover, in many instances the very typology and layout of structures (e.g. multiple walls, structures connected to each other and removal or abandonment of structures) imposed a limit on accuracy that, given the present resolution of commercially available imagery, is likely to remain to some extent intractable (see Conclusions). However, having four or more bands in the multi-spectral image did help in a few cases to distinguish between vegetation and man-made structures when the latter were constructed out of different materials, and we believe therefore that these options should always be selected when obtaining imagery. Beyond these challenges, availability of cloud free images may be a serious constraint in some locations, particularly when a very short delay between the analysis and image time point is needed (i.e. in dynamic, evolving situations): for example, in DRC we excluded several candidate sites for analysis because no cloud free images were available.

While some occupancy data were available for each site, the literature search was onerous and had a low yield. For one site (Sherkole), the sole estimate available was clearly implausible and resulted in an under-estimate of population. In general, we found very few actual structure occupancy estimates, and had to rely instead on household size figures. These were sometimes fairly divergent within the same site (see Additional file 1), and their sparsity made it difficult to construct statistically meaningful confidence intervals around the population estimates. The hierarchy of evidence for structure occupancy information that we used to attribute weights to each report (Table 2) is an attempt to rely on all information available while minimising likely bias, but criteria and scores used in this hierarchy are ultimately arbitrary and can never fully reflect the actual validity of any individual estimate. Occupancy is known to fluctuate over time, particularly in situations of protracted crisis, and thus to some extent our decision to include data from fairly remote periods may actually have increased inaccuracy (of note, sites with the highest information score did not have the greatest accuracy). It is likely that this may partly explain why sites with quality images and simple structure layouts (Farchana, Bambu) did not perform as well as expected. This limitation could be addressed by carrying out a small, rapid structure occupancy survey to provide locally appropriate data, but this is only an option if the site is accessible and diminishes the method’s relative advantages over other options. We tested such an approach in Chad (paper forthcoming).

The above drawbacks are partly a result of our choice to investigate a simple, manual method designed to empower non-specialists to carry out population estimation. Automated counting methods would necessitate a far higher skills level and thus require input by centres of excellence in remote sensing. While a review of automated or semi-automated methods is beyond the scope of this paper, this is an area of vibrant research, and we believe that these methods have a considerably larger potential for improvement than manual analysis. Automation would prove particularly valuable in scenarios where population estimates need to be updated frequently to track displacement dynamics, and could perhaps provide a solution for urban areas in which the manual method may never perform as accurately as needed.

### Study limitations

Our findings should be considered conservative, as they reflect application of the method in a more challenging set of conditions than would be the case in more current application by an agency with a recognised mandate. In prospective application of the method, it may be possible to commission new imagery, thereby ensuring a minimal time difference between the analysis and imagery time points, though again subject to constraints such as cloud cover. Contemporary sensors increasingly have wider spectral ranges, allowing various false colour combinations so as to maximise the contrast between structures and other landscape features. While costs of imagery are already reasonable, it is also likely that in future crises VHSR imagery will be reducing in cost and, in certain large scale emergencies, be available free of cost as was the case in Haiti. Photographs taken by unmanned drones or other aircraft could also be used, particularly if agencies pool together resources to obtain such images. However, it should be noted that coordination of agencies around procurement and use of satellite imagery, as well as, more broadly, sharing of resources for timely assessment and monitoring, has proven challenging in a variety of recent emergencies, and may remain so unless a clear mandate and resources are attributed to a lead agency.

In routine practice, it is likely that some real-time ground information may be available to the analyst, though data requests would have to be of limited burden to field workers (e.g. if email contact with anyone familiar with the site is possible, sample image screen-grabs could be shared with the field to help define the nature of certain areas or structures); in the early phase of displacement, it is likely that there would be few non-residential structures, thus simplifying the count; furthermore, the bank of occupancy reports available for any given site would probably be larger, as current emergencies benefit from more frequent assessments and household surveys, with a greater proportion of reports made available online; further reports could also be obtained through contact with field agencies, and the occupancy data bank could be built up progressively.

On the other hand, our results may not be fully reflective of the range of conditions found in contemporary IDP or refugee settlements. Despite a broad search, we could not identify a sufficient number of IDP, acute emergency and urban sites to analyse, mainly because of the lack of reference population estimates. A relative majority of displaced people currently are IDPs in urban settings [29]. It is possible that the choice of sites we analysed may have led to overly optimistic conclusions regarding the method’s likely performance. Our method alone would not be useful for urban sites featuring displaced populations living alongside residents: a ground survey would be needed alongside it to estimate the number of displaced occupants per structure, as discussed above.

Lastly, despite investigating several sites, this study should not be seen as providing definitive answers. In particular, due to limited resources we did not fully explore the likely extent of inter-rater reliability among different analysts: future evaluations should test counting accuracy and agreement on a larger panel of analysts with varying expertise.

## Conclusions and way forward

This study demonstrates the potential value of imagery-based population estimation to rapidly generate information on displaced and, more broadly, emergency-affected populations. Such a method is however very unlikely to be universally applicable. In particular, we believe the method has serious and potentially insurmoun limitations in urban settings, such as Port-au-Prince after the earthquake, where buildings are connected to each other, with a dense, complex pattern of roofs, and where multi-level buildings are prevalent; and in some camp settings where temporary shelters may share the same roof or tarpaulin.

The method may provide a cost-effective alternative to current options for sites with individually distinguishable structures. The method’s accuracy can probably be improved beyond that shown here. Its further application and development would benefit from the following provisions: (i) establishment of a technical unit, housed within an impartial humanitarian agency or a centre of excellence, tasked with performing rapid population estimation using manual and automated approaches,coordinate other stakeholders and advocate for more widely available imagery; (ii) research on the viability of user-friendly, open-source software application (e.g. as an extension of Google Earth or OpenStreetMap (http://www.openstreetmap.org/) enabling any online user to analyse images manually and mark structures: this would allow for crowd sourcing approaches, whereby volunteers contribute individual counts of a given site and an average is taken reflecting a broader, potentially more accurate set of counts; (iii) refinement of the structure occupancy hierarchy of evidence, with group expert consensus on criteria and sub-scores for each, e.g. based on similar exercises for mortality and nutritional surveys [30]; (iv) continued research on automated analysis methods especially around more complex or rapidly changing situations, either as stand-alone or as a complement to manual approaches.

The above improvements will require focussed, coordinated work among humanitarian relief stakeholders and continued funding for research and development. We caution however that the present method or similar approaches should only be considered an imperfect solution in the absence of adequate data from the ground: their development should not deter efforts to prioritise adequate ground assessments and measurement from the very start of a humanitarian emergency.

## Abbreviations

DRC: Democratic Republic of Congo; GIS: Geographic Information System; IDP: Internally displaced person; UNHCR: United Nations High Commissioner for Refugees; USD: United States Dollar; VHSR: Very high spatial resolution.

## Competing interests

The authors declare that they have no competing interests.

## Authors’ contributions

FC, BS and CG designed the study. FC and JP designed and carried out the occupancy search. All authors did manual analysis of satellite imagery. FC and CG analysed data and wrote the manuscript. All authors read, made substantial contributions to and approved the manuscript.

## Supplementary Material

Additional file 1 Site descriptions, detailed literature search strategy and the complete list of documents found in the search. Click here for file
