# Supplementary material for: Validity and feasibility of a satellite imagery-based method for rapid estimation of displaced populations
Source: Int J Health Geogr. 2013 Jan 23;12:4. doi: 10.1186/1476-072X-12-4 (PMC3558435; doi:10.1186/1476-072X-12-4)
Supplement: Additional file 1 — Site descriptions, detailed literature search strategy and the complete list of documents found in the search. [file 1476-072X-12-4-S1.pdf]

Validity and feasibility of a satellite imagery-based method for rapid estimation of displaced populations

Additional file

1. Narrative description of study sites

The following site descriptions mostly refer to conditions as of the date of analysis for each site.

Kutupalong refugee camp

Kutupalong refugee camp is located along the coast of southeastern Bangladesh, about 120 km from the Myanmar border in Cox's Bazar District. It is one of two remaining camps established in 1992 following an influx of 250,000 Burmese Rohingya refugees fleeing violence in Rakhine State, Myanmar [1]. While 90% were eventually repatriated [2], a fraction remained, and many more unregistered refugees arrived to Cox's Bazar over the following decade. Termed "people of concern", neither the original refugee population nor the new unregistered refugees enjoy any legal status in Bangladesh [3]. Quality of life is thus very poor because of malnutrition, insufficient housing and sanitation, poverty and lack of educational opportunities. Due to legal barriers to camp expansion, most refugees live around or adjacent to the official camp in makeshift settlements where conditions are worse than inside the camp. In Kutupalong camp itself, dwelling size has remained constant at about 9-10 square meters, regardless of the number of family members or inhabitants [2]. Roofs made of plastic sheeting frequently leak and the bamboo partitions are easily broken, leaving dwellings insecure and vulnerable to weather, especially storms and floods. Housing is cited by refugees as the second most problematic aspect of the camp, behind food.

Breidjing and Farchana refugee camps

Farchana and Breidjing refugee camps are located in eastern Chad approximately 50km from the Sudan border along the road linking the towns of El Geneina in Darfur, Sudan and Abéché in Chad. They were among seven camps established by UNHCR in 2004 to relocate refugees fleeing conflict in Darfur from their unsafe border settlements. By 2009, 260 000 Sudanese refugees were living in 12 camps in this area. Due to internal conflict in Chad, an additional 170 000 people were also living in nearby IDP sites [4], such that a quarter of the population living in the region were displaced and in need of assistance [5]. This area receives very little rainfall [4] making access to food, water, firewood and grazing land extremely scarce and a ready cause of conflict between local people and refugees [5]. Although refugees have the right to live and work in Chad [6], the vast majority live in official camps. IDPs have largely been denied the food and shelter aid given to refugees but tended to settle near these camps to take advantage of health and educational services that were superior to those locally available [7-8]. Refugees were initially given tents as emergency shelter which, after several years of use, became worn down in the desert climate [7, 9]; some families replaced these with mud-brick shelters [8] which in Farchana proved susceptible to flooding [6]. The camps resembled small towns, which was at odds with the rural semi-nomadic lifestyle most refugees led before the conflict [10].

Mugunga III & Bambu IDP camps

Bambu and Mugunga III IDP camps are located in North Kivu province, eastern Democratic Republic of Congo (DRC). Both were established as a result of regional insecurity despite a 2003 peace agreement that ended the second Congo War. Intermittent clashes along shifting battle lines created a fluid situation of continual displacement and return, establishment of new IDP camps and the abandonment of old ones. For instance, while just under a million people were estimated to be displaced in North Kivu in February 2009, in the first half of that year, 300 000 returned to newly secure areas while a further 350 000 were displaced because of insecurity [11]. With increased numbers of returns in both camps, there is a need to regularly identify and destroy 'ghost huts' of IDPs who have left and consolidate living areas [12-13].

Bambu is in Rutshuru territory, 80km north of Goma, and west of the Ugandan border. IDPs settled in By November 2008, over 25 000 IDP had gathered in Bambu town near a UN base, in hopes of protection [14-15], but food distribution did not begin until July 2009 and provision of plastic sheeting and non-food items five months after that [16].

Mugunga III is in Masisi territory, 15km west of the city of Goma on the Rwandan border. Mugunga III was created in December 2008 to relieve the burden on Mugunga I and II camps established two years earlier [17]. It is located on a hardened lava field hilltop [18-19].

Sherkole refugee camp

Located 50km from the border, Sherkole was opened by UNHCR in 1997 in response to an influx of Southern Sudanese refugees fleeing conflict in Blue Nile and Upper Nile States of Sudan [20]. During the Sudanese war, the population consisted mainly of ethnic Maban (the majority), Uduk and Funj people [21] but in 2005 they were joined by almost 3000 new refugees from Eritrea and the Great Lakes Region [22] in anticipation of a repatriation of Sudanese refugees that began in 2006 [23]. The camp overlooks ravines and foothills in a semi-arid area which supports the construction of traditional mud and thatch or mud-brick buildings [20, 22, 24]. Refugees maintained backyard gardens to supplement food rations [21, 24]. UNHCR investment in infrastructure and facilities as well as market opportunities has also led to increased settlement of host indigenous population around the camp [24].

Shimelba refugee camp

Shimelba refugee camp is located in a semi-arid region 25km from the Eritrean-Ethiopian border. It was relocated to its current location in 2004 from a more dangerous temporary site closer to the border [25] but the area is still considered one of the most highly militarized regions in the Horn of Africa [26]. The camp is in an isolated area of cleared land and low density dryland forest [25] that has been subject to environmental degredation. It hosts Eritreans fleeing a border war between 1998 and 2000, of whom the majority Tigrinya ethnic group are mainly young males from urban centres fleeing military conscription [27], while the Kunama ethnic group are agro-pastoralists [28]. The camp continues to grow and a second camp was created after Shimelba exceeded its 10 000 person capacity in 2005 [25]. Kunama people have tended to own larger plots of land than Tigrinya, living in mud and thatch housing and practicing backyard farming. Tigrinya people have tended to build mud brick houses with plastic or corrugated iron sheeting; living conditions are more crowded as new arrivals have preferred to join friends and relatives on existing plots rather than establish new ones on the camp periphery [27]. Overseas remittances have also supported the establishment of shops, salons and restaurants in the camp [29].

Champ de Mars IDP camp; Delmas 24, Sollino & Fort National neighbourhoods

Champ de Mars, Fort National, Sollino & Delmas 24 are neighbourhoods (or *quartiers*) in metropolitan Port-au-Prince, and were severely affected by an earthquake in January 2010 [30]. Unsafe building construction led to the damage or destruction of 285 000 housing units in the disaster [31], making 1.5 million homeless [32]. Around 600 000 people left the city soon after the earthquake due to scarcities of shelter, food and basic services [31] but most people constructed shelters themselves from salvaged material [33] in public spaces within their own districts (85% in same commune, 73% within same communal section) [32]. Over the following year, almost all shelters were incrementally improved to protect IDPs from heavy rains through relief distributions of plastic sheeting and basic shelter materials [33], however lack of space limited areas for cooking, agriculture and social activities [34].

The eight large IDP camps in Champ de Mars [35] occupied a 42-acre network of parks and plazas around the Presidential Palace [36-38], and were inhabited mainly by people from hilly residential areas including Fort National [34, 38]. Fort National, Sollino & Delmas 24 neighbourhoods lie on hilly ground northeast of city centre. The two sites were the first to be investigated as part of this study, and featured slightly higher-resolution imagery (0.5m) than the other sites (0.6m).

Kakuma refugee camp

Kakuma refugee camp is located in a hot, arid, semi-desert region of Turkana District, in northwest Kenya. It was established by UNHCR in 1992 in response to an influx of 23 000 Sudanese refugees fleeing conflict, including 13 000 unaccompanied boys [39], and it has continued to grow since then. Sudanese refugees continued to be the most populous residents of Kakuma in 2005 [40-41]; other large groups included refugees from Somalia and Ethiopia and it served as a destination for refugees from countries as far away as Burundi, DRC and Eritrea. The camp is situated along a dry river bed [42]. In 2006 durable shelters were scarce, with most residents living in mud brick houses or tents made of plastic sheeting and wooden poles [41]. The camp was described in 2006 as "a sprawling expanse of huts organized along tribal lines, its perimeter fenced with concertina wire, and surrounded by desert"[40], and has been cited as one of the worst examples of long-term refugee 'warehousing' [43-44], but in 2005 contained 27 schools [45], a hospital and four clinics [42]. These resources have attracted a large settlement of host Turkana population in Kakuma Town, which grew from 5000 inhabitants in 1991 [39] to 65 000 in 2006 [45].

Bairro Esturro neighbourhood

Bairro Esturro is an urban neighbourhood in Beira, the second largest city in Mozambique. Like the capital Maputo, the city can be characterised spatially as divided by the types of building materials used. An interior 'cement city' is surrounded by a larger 'cane city' which originally served as a 'dormitory' for low-cost labour in the colonial period [46]. Part of the 'cement city', Bairro Esturro saw an influx of migration shortly after Independence as Mozambicans claimed housing there abandoned by ethnic Portuguese who left the country [47]. During the war, cities like Beira were also safe havens for IDPs [48]. Originally designed to accommodate about 70 000 people, by 1991 Beira city was home to almost half a million [49], but the population of Esturro grew at a lower rate since it was already so densely inhabited [47]. More than 50% of Beira's households can be classified as 'squatters' [47].

2. Structure occupancy search strategy

The following databases and information platforms were searched:

- the Ovid platform, covering the CAB abstracts, Embase, Global Health and Medline databases, which cover scientific papers and reports in the biomedical and public health fields (Embase, Global Health and Medline) as well as agriculture, environment, veterinary sciences, applied economics, food science and nutrition (CAB abstracts), and contain peer-reviewed articles, conference abstracts and reports.
    - The full Ovid search strategy is listed below, and consisted of combinations of key words, as found in the title or abstract of the report, for (i) the site, region and country; (ii) type of data collection exercise (e.g. census, cross-sectional study, etc.); and (iii) 'household' or 'occupancy';
    - We reviewed the abstract of each report identified by the key word search, and short-listed reports that appeared to contain information on structure occupancy or household size. We then attempted to retrieve the report and reviewed the full text for eligibility;
- the Complex Emergency database (CE-DAT, http://www.cedat.be/) maintained by the Centre for Research on the Epidemiology of Disasters, which captures "humanitarian" mortality, anthropometric and/or vaccination coverage surveys shared by NGOs or UN agencies: these surveys typically estimate household size. After listing available surveys for the country in which the site analysed was located, we contacted CE-DAT to obtain electronic versions of the reports;
- the Nutrition Information in Crisis Situations (NICS, http://www.unscn.org/en/publications/nics/) database maintained by the UN Standing Committee on Nutrition: as for CE-DAT;
- the Internal Displacement Monitoring Centre (IDMC, http://www.internal-displacement.org/). We scanned the titles of documents within each country-specific library and reviewed the full-text version of those that appeared to contain occupancy information;
- the Forced Migration Online digital library (FMO, http://repository.forcedmigration.org/). After searching the library using the country as sole key term, we scanned titles and reviewed full-text reports as above;
- the Reliefweb website (www.reliefweb.int), a large clearinghouse of information for the humanitarian sector. We did both country-specific and site-specific searches using the advanced search function of the website (including analysis, assessment, appeal, evaluation and other content formats, and specifying the key terms "refugees" or "displaced" with the exception of the Haiti sites and Beira, which featured many non-displaced people), again scanning titles of search hits for potential relevance;
- the Google search engine. We did a targeted, site-specific search for .pdf, .doc and .docx files using the site name and various key terms for structure ("structure", "household", "shelter", "tent"). As even these targeted searches generally returned hundreds or thousands of hits with decreasing relevance, we only reviewed the first 10 pages of the search results;
- lastly, so as to capture pre-displacement/pre-crisis reports, we reviewed all available reports of any Multiple Indicator Cluster Surveys (MICS, http://www.childinfo.org/mics.html), Demographic and Health Surveys (DHS, http://www.measuredhs.com/) and census exercises.

3. Full Ovid platform search strategy

Example of search strategy for a country with one site (Kakuma):

1. kakuma.mp.
   Kenya.mp. or Kenya/
2. (refugee* or internally displaced or IDP*).mp. [mp=title, abstract, original title, name of substance word, subject heading word, protocol supplementary concept, rare disease supplementary concept, unique identifier]
3. 1 or 2
4. 3 and 4
5. exp epidemiologic methods/
6. nutrition surveys/ or population surveillance/ or lot quality assurance sampling/ or registries/ or cohort studies/ or cross-sectional studies/ or sampling studies/ or censuses/ or population density/ or residence characteristics/
7. (census* or survey* or registr* or enumerat* or vaccin* or immuniz* or immunis* or nutrition).mp. [mp=title, abstract, original title, name of substance word, subject heading word, protocol supplementary concept, rare disease supplementary concept, unique identifier]
8. (household or occupancy).mp. [mp=title, abstract, original title, name of substance word, subject heading word, protocol supplementary concept, rare disease supplementary concept, unique identifier]
9. or 7 or 8 or 9
10. 5 and 10

Example for multiple sites within a region (Bambu and Mugunga III):

1. (Bamb# or Mugung#).mp.
2. (North Kivu or Democratic Republic of Congo or DRC).mp. or "Democratic Republic of the Congo"/

etc. as above.

Example from urban area (Bairro Esturro, Beira and Port-au-Prince) where IDPs are mixed with host population (IDP/refugee search terms removed):

1. (Champ?-de-Mars or Delmas or Sollino or Fort National or Port au Prince).mp.
2. Haiti.mp. or Haiti/
3. 1 or 2
4. exp epidemiologic methods/
5. nutrition surveys/ or population surveillance/ or lot quality assurance sampling/ or registries/ or cohort studies/ or cross-sectional studies/ or sampling studies/ or censuses/ or population density/ or residence characteristics/
6. (census* or survey* or registr* or enumerat* or vaccin* or immuniz* or immunis* or nutrition).mp. [mp=title, abstract, original title, name of substance word, subject heading word, protocol supplementary concept, rare disease supplementary concept, unique identifier]

7. (household or occupancy).mp. [mp=title, abstract, original title, name of substance word, subject heading word, protocol supplementary concept, rare disease supplementary concept, unique identifier]
8. 4 or 5 or 6 or 7
9. 3 and 8

4. Results of the structure occupancy search

Table A1. Results of the structure occupancy search, by site. Numbers in parentheses indicate reports not accessible by the authors. Underlined numbers indicate duplicate reports.

<table>
<tr><th rowspan="2">Site name</th><th colspan="3">Ovid platform</th><th colspan="2">CE-DAT database</th><th colspan="2">Reliefweb (country-specific)</th><th colspan="2">Reliefweb (site-specific)</th><th colspan="2">Internal Displacement Monitoring Centre</th><th colspan="2">Forced Migration Online</th><th colspan="3">Google (only approximate numbers shown)</th><th>DHS, MICS, census reports</th><th rowspan="2">Total included‡</th></tr>
<tr><th>Hits</th><th>Reviewed</th><th>Included</th><th>Hits</th><th>Included</th><th>Hits</th><th>Included</th><th>Hits</th><th>Included</th><th>Hits</th><th>Included</th><th>Hits</th><th>Included</th><th>Hits</th><th>Reviewed</th><th>Included</th><th>Included</th></tr>
<tr><td>Kutupalong</td><td>14</td><td>4</td><td>0</td><td>6 (1)</td><td>4</td><td>41</td><td>0</td><td>245</td><td>0</td><td>245</td><td>0</td><td>15</td><td>0</td><td>4700</td><td>260</td><td>2</td><td>0</td><td>6</td></tr>
<tr><td>Breidjing</td><td rowspan="2">5</td><td rowspan="2">3 (1)</td><td rowspan="2">0</td><td rowspan="2">0</td><td rowspan="2">0</td><td rowspan="2">137</td><td rowspan="2">3</td><td>224</td><td><u>2</u></td><td rowspan="2">5</td><td rowspan="2">0</td><td rowspan="2">2</td><td rowspan="2">0</td><td>290</td><td>290</td><td>0</td><td>0</td><td>3</td></tr>
<tr><td>Farchana</td><td>77</td><td><u>2</u></td><td>2400</td><td>320</td><td>0</td><td>0</td><td>3</td></tr>
<tr><td>Bambu</td><td rowspan="2">23</td><td rowspan="2">8 (1)</td><td rowspan="2">2</td><td rowspan="2">7</td><td rowspan="2">4</td><td rowspan="2">612</td><td rowspan="2">1 + <u>1</u></td><td>85</td><td>0</td><td rowspan="2">168</td><td rowspan="2">1</td><td rowspan="2">34</td><td rowspan="2">0</td><td>5600</td><td>320</td><td>2</td><td>1</td><td>11</td></tr>
<tr><td>Mugunga III</td><td>379</td><td>0</td><td>1950</td><td>320</td><td>2</td><td>1</td><td>11</td></tr>
<tr><td>Sherkole</td><td rowspan="2">35</td><td rowspan="2">5 (1)</td><td rowspan="2">0</td><td rowspan="2">0</td><td rowspan="2">0</td><td rowspan="2">341</td><td rowspan="2">0</td><td>39</td><td>0</td><td rowspan="2">126</td><td rowspan="2">0</td><td rowspan="2">79</td><td rowspan="2">0</td><td>1250</td><td>280</td><td>1</td><td>0</td><td>1</td></tr>
<tr><td>Shimelba</td><td>21</td><td>0</td><td>650</td><td>280</td><td>1</td><td>0</td><td>1</td></tr>
<tr><td>Champs-de-Mars</td><td rowspan="2">781</td><td rowspan="2">21 (4)</td><td rowspan="2">2</td><td rowspan="2">5 (1)</td><td rowspan="2">2</td><td rowspan="2">708</td><td rowspan="2">1</td><td>386</td><td>0</td><td rowspan="2">0†</td><td rowspan="2">0†</td><td rowspan="2">6</td><td rowspan="2">0</td><td>106 000</td><td>410</td><td>0</td><td><u>1</u></td><td>5</td></tr>
<tr><td>Delmas 24, Sollino, Fort National</td><td>322</td><td>0</td><td>99 000</td><td>330</td><td>0</td><td><u>1</u></td><td>5</td></tr>
<tr><td>Kakuma</td><td>25</td><td>7 (1)</td><td>1</td><td>0</td><td>0</td><td>217</td><td>0</td><td>546</td><td>1</td><td>68</td><td>0</td><td>32</td><td>0</td><td>34 000</td><td>280</td><td>2</td><td>0</td><td>4</td></tr>
<tr><td>Bairro Esturro</td><td>693</td><td>80 (25)</td><td>6</td><td>0†</td><td>0†</td><td>376</td><td>0</td><td>569</td><td>0</td><td>0†</td><td>0†</td><td>31</td><td>0</td><td>22 600</td><td>260</td><td>1</td><td>0</td><td>7</td></tr>
</table>

† Country not included in the database. ‡ Included reports from country-specific searches are added to the total for each site within that country.

5. Reports included in the structure occupancy review

Table A2. List of reports included in analysis.

Country	Site	Reference	Search method	Site(s) covered by the report	Dates of data collection	Type of report	Type of estimate	Mean occupancy	Information score
Bangladesh	Kutupalong	[50]	CE-DAT	Makeshift camp surrounding official Kutupalong camp	Mar 2009	Household survey (mortality, nutritional status)	Household size (sleeping under the same roof)	5.3	384
Bangladesh	Kutupalong	[51]	CE-DAT	Kutupalong and Nayapara camps	Apr 2009	Household survey (nutritional status)	Household size (other)	6.6	71
Bangladesh	Kutupalong	[52]	CE-DAT	Kutapalong camp	Mar-Apr 2007	Household survey (nutritional status)	Household size (other)	7.0	60
Bangladesh	Kutupalong	[53]	CE-DAT	Kutupalong and Nayapara camps	Nov-Dec 2005	Household survey (nutritional status)	Household size (other)	4.8	24
Bangladesh	Kutupalong	[2]	Google	Kutupalong camp	Jan 2002	Household survey (basic needs)	Structure occupancy	8.3	240
Bangladesh	Kutupalong	[3]	Google	Kutupalong camp	2007	Programme review	Household size (other)	6.0	10
Chad	Breidjing	[54]	Reliefweb	Refugee camps in Cariari and Bahai, Eastern Chad	Jun 2004	Household survey (basic needs)	Household size (sleeping under the same roof)	6.0	384
Chad	Breidjing	[54]	Reliefweb	Refugee camps in Iridimi, Touloum and Kounoungo, Eastern Chad	Jul 2004	Household survey (basic needs)	Household size (sleeping under the same roof)	6.0	384
Chad	Breidjing	[55]	Reliefweb	Farchana camp	May 2004	Food security site assessment	Household size (other)	6.0	32
Chad	Farchana	[54]	Reliefweb	Refugee camps in Cariari and Bahai, Eastern Chad	Jun 2004	Household survey (basic needs)	Household size (sleeping under the same roof)	6.0	384
Chad	Farchana	[54]	Reliefweb	Refugee camps in Iridimi, Touloum and Kounoungo, Eastern Chad	Jul 2004	Household survey (basic needs)	Household size (sleeping under the same roof)	6.0	384

Country	Site	Reference	Search method	Site(s) covered by the report	Dates of data collection	Type of report	Type of estimate	Mean occupancy	Information score
Chad	Farchana	[55]	Reliefweb	Farchana camp	May 2004	Food security site assessment	Household size (other)	6.0	40
DRC	Bambu	[56]	CE-DAT	12 mixed host and IDP non-camp settlements in Mweso Health zone, North Kivu	Apr 2008	Household survey (mortality, nutritional status)	Household size (other)	5.3	12
DRC	Bambu	[57]	CE-DAT	21 mixed host and IDP non-camp settlements in Pinga Health Zone, North Kivu	Mar 2008	Household survey (mortality, nutritional status)	Household size (other)	6.0	12
DRC	Bambu	[58]	CE-DAT	9 mixed host and IDP non-camp settlements in Rutshuru Health Zone, North Kivu	Aug 2008	Household survey (mortality, nutritional status)	Household size (other)	6.0	12
DRC	Bambu	[59]	CE-DAT	18 mixed host (92%) and IDP (8%) non-camp settlements in Kirotshe Health Zone, Masisi Territory, North Kivu	Feb 2008	Survey (nutritional & mortality)	Household size (other)	5.9	12
DRC	Bambu	[60]	DHS	Rural DRC	2007	DHS household survey	Household size (other)	5.2	16
DRC	Bambu	[61]	Google	Seven villages in Nyiragongo territory outside Goma city, North Kivu	Oct 2008	Household survey (basic needs)	Household size (other)	5.8	2
DRC	Bambu	[62]	Google	Bambu IDP camp	Jul 2008	Household survey (mortality, nutritional status, vaccination coverage)	Household size (other)	4.3	80

Country	Site	Reference	Search method	Site(s) covered by the report	Dates of data collection	Type of report	Type of estimate	Mean occupancy	Information score
DRC	Bambu	[63]	IDMC	Eastern DRC as a whole	2009	Programme review	Household size (other)	5.0	2
DRC	Bambu	[64]	Ovid	Tché IDP camp in Ituri region, northeast DRC	Mar 2005	Household survey (mortality)	Structure occupancy	4.9	192
DRC	Bambu	[65]	Ovid	3 towns, all with mixed host population and IDP camps, in North Kivu (Kabizo, Masisi and Kitchanga)	May 2009	Household survey (mortality, access to health care)	Household size (other)	5.6	16
DRC	Bambu	[66]	Reliefweb	Non-displaced people in North Kivu, South Kivu and Ituri region	Sep-Dec 2007	Household survey (human rights violations)	Household size (sleeping under the same roof)	6.8	96
DRC	Mugunga III	[56]	CE-DAT	12 mixed host and IDP non-camp settlements in Mweso Health zone, North Kivu	Apr 2008	Household survey (mortality, nutritional status)	Household size (other)	5.3	12
DRC	Mugunga III	[57]	CE-DAT	21 mixed host and IDP non-camp settlements in Pinga Health Zone, North Kivu	Mar 2008	Household survey (mortality, nutritional status)	Household size (other)	6.0	12
DRC	Mugunga III	[58]	CE-DAT	9 mixed host and IDP non-camp settlements in Rutshuru Health Zone, North Kivu	Aug 2008	Household survey (mortality, nutritional status)	Household size (other)	6.0	12
DRC	Mugunga III	[59]	CE-DAT	18 mixed host (92%) and IDP (8%) non-camp settlements in Kirotshe Health Zone, Masisi Territory, North	Feb 2008	Household survey (mortality, nutritional status)	Household size (other)	5.9	12

Country	Site	Reference	Search method	Site(s) covered by the report	Dates of data collection	Type of report	Type of estimate	Mean occupancy	Information score
				Kivu					
DRC	Mugunga III	[60]	DHS	Rural DRC	2007	DHS household survey	Household size (other)	5.2	16
DRC	Mugunga III	[61]	Google	Seven villages in Nyiragongo territory outside Goma city, North Kivu	Oct 2008	Household survey (basic needs)	Household size (other)	5.8	2
DRC	Mugunga III	[62]	Google	Bambu IDP camp	Jul 2008	Household survey (mortality, nutritional status, vaccination coverage)	Household size (other)	4.3	64
DRC	Mugunga III	[63]	IDMC	Eastern DRC as a whole	2009	Programme review	Household size (other)	5.0	2
DRC	Mugunga III	[64]	Ovid	Tché IDP camp in Ituri region, northeast DRC	Mar 2005	Household survey (mortality)	Structure occupancy	4.9	192
DRC	Mugunga III	[65]	Ovid	3 towns, all with mixed host population and IDP camps, in North Kivu (Kabizo, Masisi and Kitchanga)	May 2009	Household survey (mortality, access to health care)	Household size (other)	5.6	16
DRC	Mugunga III	[66]	Reliefweb	Non-displaced people in North Kivu, South Kivu and Ituri region	Sep-Dec 2007	Household survey (human rights violations)	Household size (sleeping under the same roof)	6.8	96
Ethiopia	Shimelba	[25]	Google	Shimelba camp	2006	Household survey (fuel consumption)	Household size (other)	4.7	60
Ethiopia	Sherkole	[67]	Google	Sherkole camp	Jul 2003	Site assessment	Household size (other)	3.1	16

Country	Site	Reference	Search method	Site(s) covered by the report	Dates of data collection	Type of report	Type of estimate	Mean occupancy	Information score
Haiti	Champs de Mars	[68]	CE-DAT	5 urban slum areas of Port-au-Prince	Aug 2007	Household survey (mortality, nutritional status)	Household size (other)	5.2	32
Haiti	Champs de Mars	[69]	DHS	Metropolitan stratum, Haiti	2005-2006	DHS household survey	Household size (other)	4.3	8
Haiti	Champs de Mars	[70]	Ovid	Greater Port-au-Prince	Dec 2005	Household survey (human rights violations)	Household size (other)	4.5	16
Haiti	Champs de Mars	[71]	Ovid	GHESKIO IDP camp, Port-au-Prince	Feb 2010	Programme review	Structure occupancy	5.3	800
Haiti	Champs de Mars	[72]	Reliefweb	Cite Soleil slum, Port-au-Prince	2006	Household survey (mortality, violence)	Household size (other)	5.0	8
Haiti	Delmas 24, Sollino, Fort National	[68]	CE-DAT	5 urban slum areas of Port-au-Prince	Aug 2007	Household survey (mortality, nutritional status)	Household size (other)	5.2	32
Haiti	Delmas 24, Sollino, Fort National	[69]	DHS	Metropolitan stratum, Haiti	2005-2006	DHS household survey	Household size (other)	4.3	8
Haiti	Delmas 24, Sollino, Fort National	[70]	Ovid	Greater Port-au-Prince	Dec 2005	Household survey (human rights violations)	Household size (other)	4.5	16
Haiti	Delmas 24, Sollino, Fort National	[71]	Ovid	GHESKIO IDP camp, Port-au-Prince	Feb 2010	Programme review	Structure occupancy	5.3	800
Haiti	Delmas 24, Sollino, Fort National	[72]	Reliefweb	Cite Soleil slum, Port-au-Prince	2006	Household survey (mortality, violence)	Household size (other)	5.0	8
Kenya	Kakuma	[73]	Ovid	Dadaab refugee camp, eastern Kenya	Apr-Aug 2002	Survey (baseline for vector control intervention)	Household size (sleeping under the same roof)	6.6	144
Kenya	Kakuma	[74]	Google	Kakuma camp	Sep 2002	Survey (nutritional status)	Household size (other)	8.2	64

Country	Site	Reference	Search method	Site(s) covered by the report	Dates of data collection	Type of report	Type of estimate	Mean occupancy	Information score
Kenya	Kakuma	[75]	Google	Kakuma camp	2005	Survey (environmental health)	Household size (other)	6.7	60
Kenya	Kakuma	[76]	Reliefweb	Kakuma camp	2000	Programme review	Structure occupancy	2.5	160
Mozambique	Bairro Esturro	[77]	Google	Beira city	Aug 2002	Household survey (crime and police victimisation)	Household size (other)	6.0	60
Mozambique	Bairro Esturro	[78]	Ovid	Maputo city	Sep 2000	Household survey (fuel consumption)	Household size (other)	5.9	24
Mozambique	Bairro Esturro	[79]	Ovid	Bairro Mucessua, Vila Gondola town, Manica Province	Jun 1993-May 1995	Household survey (ethnography of pregnancy)	Household size (other)	7.0	24
Mozambique	Bairro Esturro	[80]	Ovid	Urban Mozambique (large urban areas only)	Feb 1996- Apr 1997	National household survey of living conditions (poverty)	Household size (other)	6.4	32
Mozambique	Bairro Esturro	[81]	Ovid	Maputo	Aug 1995-Apr 1997	Case-control study (Vitamin A supplementation)	Household size (other)	6.0	24
Mozambique	Bairro Esturro	[82]	Ovid	Maputo	not reported	Case-control study (painful pregnancies)	Household size (other)	7.0	24
Mozambique	Bairro Esturro	[83]	Ovid	Maputo	Feb-Mar 2003	Household survey (mental health)	Household size (other)	6.1	64

6. References

1. Helen Keller International: **Annual Nutrition Survey, Rohingya Refugee Camp, Cox's Bazar, Bangladesh**. In. Cox's Bazar: HKI; 2009.
2. Medecins Sans Frontieres Holland: **10 YEARS FOR THE ROHINGYA REFUGEES IN BANGLADESH: PAST, PRESENT AND FUTURE**. In. Amsterdam: MSF; 2002.
3. United Nations High Commissioner for Refugees: **Bangladesh: Analysis of Gaps in the Protection of Rohingya Refugees**. In. Geneva: UNHCR; 2007.
4. SSA: **Case study of sustainable sanitation projects: household pit latrines with urine diversion: Farchana refugee camp, Chad**. In.: Sustainable sanitation alliance; 2011.
5. WFP-UNHCR: **Joint assessment mission (JAM) 2008: The humanitarian crisis created by the displacement of Sudanese refugees and internally-displaced persons in Eastern Chad, September 9-17, 2008** In.: World Food Programme/United Nations High Commissioner of Refugees; 2008.
6. USCRI: **World Refugee Survey 2008 - Chad**. In.: United States Committee for Refugees and Immigrants; 2008.
7. Herz M: **Research paper no 147: Refugee camps in Chad: planning strategies and the architect's involvement in the humanitarian dilemma**. In: *New issues in refugee research.* UNHCR; 2007.
8. Michael M, Pearson N, Daliam A: **Interagency health evaluation: Humanitarian oasis in a parched health sector: refugees and host populations in Eastern and Southern Chad, conducted February 2006**. In.: Core Working Group for Inter-agency Health Evaluations; 2006.
9. STAND: **Camp Farchana**. In.: STAND; Undated.
10. IFRC/RCS: **International Federation of Red Cross and Red Crescent Societies (2005) Annual Report** In.: International Federation of Red Cross and Red Crescent Societies; 2005.
11. UNHCR: **UNHCR and UNOPS partner to address IDP 'real needs' in Congo's east**. In.: UN High Commissioner for Refugees, UN Office for Project Services; 2009.
12. PU: **Rapport de gestion des camps de la zone de sante de Birambizo, Avril 2010**. In.: Première Urgence; 2010.
13. DC4IDP: **Republic Democratique du Congo, Province du Nord Kivu: Camp IDPs de Mugunga III/Ville de Goma, Aout 2010**. In.: Data Centre for IDP Population, UNOPS, UNHCR; 2010.
14. Prior M: **Fear and desperation in North Kivu**. In.: World Food Programme; 2008.
15. OCHA: **DR Congo Humanitarian Situation in North Kivu – no 13 Situation Report, 14 to 17 November 2008**. In.: UN Office for the Coordinatiom of Humanitarian Affairs; 2008.
16. UNHCR: **Qui fait quoi ou: CCCM Zone de Sante Birambizo**. In.: United Nations High Commissioner for Refugees, Premiere Urgence; 2010.
17. UNHCR: **Situation in Democratic Republic of the Congo, Briefing Notes, 8 May 2009**. In.: United Nations High Commissioner for Refugees; 2009.
18. UNHCR: **Rising tensions in North Kivu push more Congolese from their homes, News Stories, 3 September 2007**. In.: United Nations High Commissioner for Refugees; 2007.
19. UNHCR: **Congolese victims of sexual violence call for help from the international community, News Stories, 16 March 2011**. In.: United Nations High Commissioner for Refugees; 2011.

20. James W: **Journeys of transformation on the Sudan-Ethiopia border**. In: *Changing identifications and alliances in north-east Africa, Volume II: Integration and conflict studies.* Edited by Schlee G, Watson E: Berghahn Books; 2009.

21. UNHCR: **2003 Joint Assessment Mission**. In.: UNHCR/ARRA/WFP; 2003.

22. UNHCR: **UNHCR Global Report 2005 - Ethiopia**. In.: United Nations High Commissioner for Refugees; 2005.

23. Gebresilassie T: **Engendering 'voluntary' repatriation: the planned repatriation of Maban refugees from Sherkole refugee camp, Ethiopia, to South Sudan**. Budapest: Central European University; 2007.

24. Fetene G: **Refugees impact on woodland resources: the case of Sherkole refugee camp**. Addis Ababa: Addis Ababa University; 2009.

25. Egziabher A, Murren J, O'Brien C: **An ethanol-fueled household energy initiative in the Shimelba Refugee Camp, Tigray, Ethiopia: a joint study by the UNHCR and the Gaia Association**. In.: UNHCR & Gaia Association; 2006.

26. COR: **COR Centre refugee backgrounder no. 5: Eritrean refugees from Shimelba refugee camp**. In.: Cultural Orientation Resource Centre, Centre for Applied Linguistics; 2010.

27. UNHCR: **Pilot project proposal on multi-story garden: Shimbela refugee camp**. In.: United Nations High Commissioner for Refugees; 2008.

28. COR: **COR Centre refugee backgrounder no. 3: The Kunama**. In.: Cultural Orientation Resource Centre and Centre for Applied Linguistics; 2007.

29. COR/JVA: **Eritrean refugees in the Shimelba refugee camp, Ethiopa**. In.: Cultural Orientation Resource Centre & Joint Voluntary Agency, Church World Service; Undated.

30. CBC: **Haiti raises quake death toll on anniversary**. In.: Canadian Broadcasting Corporation; 2011.

31. Kelly C, Solberg S: **Rapid Environmental Impact Assessment: Haiti Earthquake - January 12, 2010**. In.: USAID Haiti; 2010.

32. CCCM-Haiti: **Registration of internally displaced populations affected by the earthquake in Haiti: Phase 1: Emergency registration, Final report Dec 2010**. In.: Camp Coordination & Camp Management Cluster, Haiti; 2010.

33. IASC: **Emergency shelter Haity: Field notes April 2011**. In.: Inter-Agency Standing Committee, Shelter Cluster; 2011.

34. OCHA: **Haiti earthquake situation report #25, 1 March 2010**. In.: United Nations Office for the Coordination of Humanitarian Affairs; 2010.

35. MSF: **Système de surveillance de l'état de santé de la population, Quartier de Champs de Mars, Haiti. Census and Vaccination Coverage Report, Week 16, 19 – 24th April 2010**. In.: Medecins Sans Frontiere, Epicentre; 2010.

36. Grimm F: **In Haiti's tent cities, a return to normalcy is unimaginable**. In.: Miami Herald, 12 July 2010; 2010.

37. Husarka A: **Historic Park in Haiti's Capital Overflows with Quake Survivors**. In.: International Rescue Committee, 26 Jan 2010; 2010.

38. Sontag D: **In Haiti, the displaced are left clinging to the edge**. In.: New York Times; 2010.

39. Jamal A: **Minimum standards and essential needs in a protracted refugee situation: a review of the UNHCR programme in Kakuma, Kenya**. In.: United Nations High Commissioner for Refugees (UNHCR); 2000.

40. Morse D: **Murder from Darfur to Cairo**. In.: Salon.com; 2006.

41. Callanan A: **Joint assessment mission, Kenya, 18 September - 6 October 2006**. In.: WFP/UNHCR/GoK/Donors; 2006.

42. Bayoh MN, Akhwale W, Ombok M, Sang D, Engoki SC, Koros D, Walker ED, Williams HA, Burke H, Armstrong GL *et al*: **Malaria in Kakuma refugee camp, Turkana, Kenya: facilitation of Anopheles arabiensis vector populations by installed water distribution and catchment systems**. *Malar J* 2011, **10**:149.

43. Smith M: **Warehousing refugees: a denial of rights, a waste of humanity**. In: *World Refugee Survey 2004.* World Refugee Survey; 2004.

44. James-Deramo M: **Perceptions of aid organizations in Kakuma refugee camp**. In.: Kakuma News Reflector – A Refugee Free Press; 2011.

45. Jansen B: **The accidental city: urbanisation in an East-African refugee camp**. *Urban Agriculture magazine* 2009(21):11-12.

46. CEDH: **Mozambique, Cities Without Slums, Analysis of the Situation & Proposal of Intervention Strategies**. In. Maputo: Center for Habitat Studies and Development, Center for Habitat Studies and Development; 2006.

47. Elmes G, Koti F, Lin G, Masilela C, McCusker B, Weiner D, Jose F, Schultheis M, Correia J, Saxon G *et al*: **Monitoring Beira using geographic urban indicators, Final report: Year one activities**. In.: Dept Geology & Geography, West Virginia University, Catholic University of Mozambique; Undated.

48. UNDP: **Overview of UNDP's involvement in the reintegration of IDPs and returnees in post-conflict contexts**. In.: United Nations Development Programme; Undated.

49. Collins C: **Mozambique's HIV/AIDS pandemic: Grappling with apartheid's legacy**. In: *Social Policy and Development Programme Paper Number 24, Feb 2006.* United Nations Research Institute for Social Development; 2006.

50. Medecins Sans Frontieres Holland: **Cross sectional nutrition, retrospective mortality and measles vaccination coverage survey, Kutupalong Makeshift Camp, 18th to 24th March 2009**. In. Amsterdam: MSF; 2009.

51. Helen Keller International: **Annual Nutrition Survey, Rohingya Refugee, Cox's Bazar, Bangladesh**. In. Cox's Bazar: HKI; 2009.

52. Helen Keller International: **Report on assessment of malnutrition and micronutrient status of children and pregnant women living in the Rohingya Refugee Camps**. In. Dhaka: HKI; 2007.

53. United Nations High Commissioner for Refugees, National Institute of Research For Food And Nutrition: **Mortality and Nutrition Survey in Nayapara and Kutupanlong Camps**. In. Dhaka: UNHCR; 2006.

54. Tomczyk B, Dunne E, Chang M, Fedele S, Talley LE, Blanton C: **Emergency nutrition and mortality surveys conducted among Sudanese refugees and Chadian villagers, northeast Chad, June 2004**. In. Atlanta: US Centers for Disease Control. http://www.cdc.gov/globalhealth/gdder/ierh/ResearchandSurvey/Chad_report04.pdf (accessed 25 July 2012). 2004.

55. LeJeune S: **Rapid household economy assessment Farchana refugee camp Eastern Chad, Final report**. In.: Save the Children; 2004.

56. Cooperazione Internazionale: **Rapport Enquete Nutritionnelle: Anthropometrie et mortalite realisee dans la Zone de Sante de Mweso (Province Du Nord Kivu), R D Congo**. In. Goma: Coopi; 2008.

57. Cooperazione Internazionale: **Rapport Enquete Nutritionnelle: Anthropometrie et mortalite, la Zone de Sante de Pinga, Territoire de Walikale (Province Du Nord Kivu), R D Congo**. In. Goma: Coopi; 2008.

58. Cooperazione Internazionale: **Rapport Enquete Nutritionnelle: Anthropometrie et mortalite realisee dans la Zone de Sante de Rutshuru (Province Du Nord Kivu), R D Congo**. In. Goma: Coopi; 2008.

59. World Vision: **Rapport Enquete Nutritionnelle: Anthropometrie et mortalite, Zone de Sante de Kirotshe (Province Du Nord Kivu)**. In. Goma: World Vision; 2008.

60. Ministère du Plan and Macro International: **Enquête Démographique et de Santé, République Démocratique du Congo 2007**. In. Calverton, Maryland, USA: Ministère du Plan and Macro International; 2008.

61. Mercy Corps: **Baseline Survey, Water, Sanitation and Hygiene: Nyiragongo Territory, North Kivu Province, Democratic Republic of the Congo (DRC), October 2008**. In. Goma: Mercy Corps. http://pdf.usaid.gov/pdf_docs/PNADR869.pdf (accessed 26 July 2012). 2008.

62. Grellety E, Ronsse A: **Enquête de mortalité rétrospective, anthropométrique et de couverture vaccinale rougeole dans le camp de déplacés de Bambu & Mise en place d'un système de surveillance dans les camps de déplacés de Bambu, Kasoko et Katsiru. Nyanzale Zone de santé de Biram**. In. Paris: Epicentre; 2008.

63. United Nations Office for Coordination of Humanitarian Affairs: **Population movements in eastern DR Congo, October-December 2009**. In. Kinshasa: OCHA. http://reliefweb.int/sites/reliefweb.int/files/resources/F3D219BC4E418152852576DB00601798-Full_Report.pdf (accessed 31 Jul 2012); 2009.

64. Ahoua L, Tamrat A, Duroch F, Grais RF, Brown V: **High mortality in an internally displaced population in Ituri, Democratic Republic of Congo, 2005: results of a rapid assessment under difficult conditions**. *Glob Public Health* 2006, **1**(3):195-204.

65. Alberti KP, Grellety E, Lin YC, Polonsky J, Coppens K, Encinas L, Rodrigue MN, Pedalino B, Mondonge V: **Violence against civilians and access to health care in North Kivu, Democratic Republic of Congo: three cross-sectional surveys**. *Confl Health* 2010, **4**:17.

66. Vinck P, Pham P, Baldo S, Shigekane R: **Living with Fear: A population-based survey on attitudes about peace, justice, and social reconstruction in Eastern Democratic Republic of Congo**. In. Berkeley: Human Rights Center, University of California at Berkeley. http://www.law.berkeley.edu/HRCweb/pdfs/LivingWithFear-DRC.pdf (accessed 24 Jul 2012). 2008.

67. Administration for Refugee and Returnee Affairs, United Nations High Commissioner for Refugees, World Food Programme: **2003 Joint Assessment Mission**. In. Addid Abeba: ARRA; 2003.

68. Concern Worldwide: **Nutrition Survey Results Summary: St. Martin, Decayette, Jalousie, bois Marquette and cite okay neighbourhoods, Port au Prince**. In. Port au prince: Concern; 2007.

69. Cayemittes M, Placide MF, Mariko S, Barrère B, Sévère B, Canez A: **Enquête Mortalité, Morbidité et Utilisation des Services, Haïti, 2005-2006**. In. Calverton, Maryland, USA: Ministère de la Santé Publique et de la Population, Institut Haïtien de l'Enfance and Macro International Inc. ; 2007.

70. Kolbe AR, Hutson RA: **Human rights abuse and other criminal violations in Port-au-Prince, Haiti: a random survey of households.[Erratum appears in Lancet. 2007 Feb 3;369(9559):370]**. *Lancet* 2006, **368**(9538):864-873.

71. Pape JW, Deschamps MM, Ford H, Joseph P, Johnson Jr WD, Fitzgerald DW: **The GHESKIO refugee camp after the earthquake in Haiti - Dispatch 2 from Port-au-Prince**. *N Engl J Med* 2010, **362**(9):e27.

72. Ponsar F, Ford N, Van Herp M, Mancini S, Bachy C: **Mortality, violence and access to care in two districts of Port-au-Prince, Haiti**. *Confl Health* 2009, **3**:4.

73. Kimani EW, Vulule JM, Kuria IW, Mugisha F: **Use of insecticide-treated clothes for personal protection against malaria: a community trial**. *Malaria Journal* 2006, **5**:63.

74. Rio D: **Nutritional survey, children 6-59 months, Kakuma Refugee Camp, Kenya**. In. Nairobi: International Rescue Committee; 2002.

75. Snuggs J: **Kakuma Refugee Camp Basic Environmental Health Survey**. In. Nairobi: International Rescue Committee; 2005.

76. United Nations High Commissioner for Refugees: **Minimum standards and essential needs in a protracted refugee situation: A review of the UNHCR programme in Kakuma,Kenya**. In. Geneva: UNHCR. http://www.unhcr.org/3ae6bd4c0.pdf (accessed 24 Jul 2012); 2000.

77. Population Studies Center EMU: **Strategic Plan of the Police of the Republic of Mozambique; Results of surveys on victimisation and police performance**. In. Maputo: UNICRI. http://rechten.uvt.nl/icvs/StratPlanPRM/ (accessed 26 July 2012). 2002.

78. Brouwer R, Falcao MP: **Wood fuel consumption in Maputo, Mozambique**. *Biomass and Bioenergy* 2004, **27**(3):233-245.

79. Chapman RR: **Endangering safe motherhood in Mozambique: prenatal care as pregnancy risk**. *Soc Sci Med* 2003, **57**(2):355-374.

80. Simler KR, Sanjukta M, Dava GL, Datt G: **Rebuilding after war: micro-level determinants of poverty reduction in Mozambique**. In: *Research Report - International Food Policy Research Institute.* Washington: International Food Policy Research Institute; 2004: xii + 96.

81. Julien MR, Gomes A, Varandas L, Rodrigues P, Malveiro F, Aguiar P, Kolsteren P, Stuyft P, Hildebrand K, Labadarios D *et al*: **A randomized, double-blind, placebo-controlled clinical trial of vitamin A in Mozambican children hospitalized with nonmeasles acute lower respiratory tract infections**. *Trop Med Int Health* 1999, **4**(12):794-800.

82. Osman NB, Folgosa E, Bergstrom S: **An incident case-referent study of threatening preterm birth and genital infection**. *J Trop Pediatr* 1995, **41**(5):267-272.

83. Patel V, Simbine APF, Soares IC, Weiss HA, Wheeler E: **Prevalence of severe mental and neurological disorders in Mozambique: a population-based survey**. *Lancet* 2007, **370**(9592):1055-1060.
